# From policy to practice: implementation of physical activity and food policies in schools

**DOI:** 10.1186/1479-5868-10-71

**Published:** 2013-06-03

**Authors:** Louise C Mâsse, Daniel Naiman, Patti-Jean Naylor

**Affiliations:** 1 School of Population and Public Health, University of British Columbia, F508-4480 Oak Street, Vancouver, BC V6H3V4, Canada; 2 School of Exercise Science, Physical and Health Education, University of Victoria, Vancouver, BC, Canada

**Keywords:** Physical education, Physical activity, Nutrition, School policies, School guidelines, Implementation, Uptake, Barriers, Facilitators, Qualitative

## Abstract

**Purpose:**

Public policies targeting the school setting are increasingly being used to address childhood obesity; however, their effectiveness depends on their implementation. This study explores the factors which impeded or facilitated the implementation of publicly mandated school-based physical activity and nutrition guidelines in the province of British Columbia (BC), Canada.

**Methods:**

Semi-structured interviews were conducted with 50 school informants (17 principals - 33 teacher/school informants) to examine the factors associated with the implementation of the mandated Daily Physical Activity (DPA) and Food and Beverage Sales in Schools (FBSS) guidelines. Coding used a constructivist grounded theory approach. The first five transcripts and every fifth transcript thereafter were coded by two independent coders with discrepancies reconciled by a third coder. Data was coded and analysed in the NVivo 9 software. Concept maps were developed and current theoretical perspectives were integrated in the later stages of analysis.

**Results:**

The Diffusion of Innovations Model provided an organizing framework to present emergent themes. With the exception of triability (not relevant in the context of mandated guidelines/policies), the key attributes of the Diffusion of Innovations Model (relative advantage, compatibility, complexity, and observability) provided a robust framework for understanding themes associated with implementation of mandated guidelines. Specifically, implementation of the DPA and FBSS guidelines was facilitated by perceptions that they: were relatively advantageous compared to status quo; were compatible with school mandates and teaching philosophies; had observable positive impacts and impeded when perceived as complex to understand and implement. In addition, a number of contextual factors including availability of resources facilitated implementation.

**Conclusions:**

The enactment of mandated policies/guidelines for schools is considered an essential step in improving physical activity and healthy eating. However, policy makers need to: monitor whether schools are able to implement the guidelines, support schools struggling with implementation, and document the impact of the guidelines on students’ behaviors. To facilitate the implementation of mandated guidelines/policies, the Diffusion of Innovations Model provides an organizational framework for planning interventions. Changing the school environment is a process which cannot be undertaken solely by passive means as we know that such approaches have not resulted in adequate implementation.

## Background

Schools provide the best setting to support a population-based approach to improve physical activity (PA) and healthy eating (HE) for all children regardless of their ethnic or socio-demographic background [1,2]. School-based public policies are increasingly being used to address childhood obesity [2,3] and emerging evidence supports the effectiveness of such policies on positively influencing the school environment. For example, school-based physical education (PE) policies have increased the amount of PE offered in schools (i.e., total minutes or days/week) [4,5] and school-based nutrition policies have resulted in less access to sugar-sweetened beverages and low nutrient energy dense foods in schools [6-8]. School-based policies also influence student behaviors. For example such policies have increased PA levels, although one study documented a greater effect for girls [4,9]; increased fitness levels [10]; and reduced student consumption of sugar-sweetened beverages in school [11-13]. Furthermore, evidence suggests that improvements to both PA and HE will result in improvements in cognitive functioning and overall academic performance, helping schools meet their academic mandate [14-16].

While the emerging evidence suggest school-based policies can positively influence the school environment and student behaviours, the impact on student body mass index (BMI) is less clear. Some studies have found no association between school-based policies and student BMI [9,11] while others documented an association [17,18]. Specifically, students had lower BMIs in states with stricter PE and school food policies [17] and in states with stronger competitive food policies [18]. The effectiveness of school-based policies against childhood obesity depends on their implementation, which is often less than optimal even when these policies are publicly mandated [4,9,10]. To date, few studies have examined the factors that impede or facilitate the implementation of school-based obesity prevention policies. Factors found to impede implementation include: potential loss of school revenues, competing curriculum demands and priorities, lack of resources (staff, funding, availability of programs or teaching resources), lack of coordination, no dedicated funding to support the mandate, industry lobbying, and misconceptions about the types of food and beverages made available at school [5,19-24]. In contrast implementation is enabled when there is support from key politicians, parents, physicians and school personnel as well as the provision of financial support and having data to inform policy decisions enabled implementation [19-22].

In Canada, the Constitution Act declares education to be under provincial jurisdiction [25], as a result any guidelines/policies affecting the school system are enacted at the provincial level. The enactment of the Daily PA (DPA) and Food and Beverage Sales in Schools (FBSS) guidelines in the province of British Columbia (BC), Canada represented the province’s first attempt to govern the PA and food environment of schools. The DPA guidelines set the requirements for daily physical activity for students. The FBSS guidelines set out minimum nutrition standards for food and beverages sold to students. In addition, both policies set out to encourage the development of life-long healthy behaviours.

Prior to the implementation of the DPA guidelines, K to 9 grades in BC were expected to devote 10% of their instructional time to PE, for grade 10 students PE was and remains a graduation requirement and for grades 11 and 12 it was and remains as an elective course [26]. The new DPA guidelines require schools to offer 30 minutes per day of PA as part of the grade K-9 educational curriculum. Grade 10-12 students must document and report a minimum of 150 minutes per week of PA, performed at a moderate to vigorous intensity. While some provinces in Canada mandate that PE be taught by a PE specialist, in BC this differs by grade. At the elementary school level PE is primarily taught by classroom teachers. Implementation of the guidelines was expected by the beginning of the 2008/2009 school year.

Prior to the implementation of the FBSS guideline, schools in BC were not expected to meet any nutritional guidelines. The FBSS guidelines were designed to maximize students’ access to healthier options and fully eliminate the sale of unhealthy food and beverages in BC schools. The FBSS guidelines mandate schools to adhere to the 2007 Canada’s Food Guide [27] for all food and beverage sold or made available at school. Full implementation of the FBSS guidelines were expected by the end of the 2007/2008 school year. While elementary schools in BC have fewer permanent food outlets than in middle/high schools (45% versus 95% of schools have permanent food outlets), less healthy food choices were often offered in elementary schools through parents or fundraising efforts (e.g., pizza lunches or hot dog days) [28].

This study explores the factors that impeded or facilitated the implementation of publicly mandated school-based PE and nutrition guidelines in the province of BC. Our study provides a unique opportunity to examine the implementation of the FBSS guidelines in a jurisdiction that did not previously have any guidelines or mandated policies to govern the food environment of schools. In addition, it also provides the opportunity to examine the implementation of a guideline that addresses the PA environment in schools while previous research conducted in other jurisdictions has focused on the implementation of policies directed at changing the PE environment in schools.

## Methods

We examined the factors associated with the implementation of the DPA and FBSS guidelines qualitatively by conducting semi-structured interviews with 50 school informants (17 principals and 33 teacher/school informants). The study protocol was approved by the University of British Columbia and the University of Victoria Research Ethics Boards and participating school districts.

### Sample selection and recruitment

Schools were selected to participate in this study based on responses to a school survey administered in the 2007-2008 school year. School selection ensured representation across: self-reported levels of implementation of the guidelines, school types (elementary, middle, and high schools), and school settings (urban, suburban, and rural). Of the 513 schools that completed the 2007-2008 school survey, a total of 47 schools were invited to participate in the qualitative study. We targeted more middle/high schools (45% versus 21%) and more schools in neighborhoods with a higher percentage of visible minorities (32% versus 20%) in our sample. This ensured an adequate representation of these perspectives in our data. By including more middle/high schools, which typically have more students and teachers than elementary schools, our targeted sample included a higher percentage of schools with more students and teachers.

Principals in selected schools received an invitational letter through mail and follow-up reminders. In total, 17 schools participated in the qualitative study (36% response rate): 10 elementary schools (grades 7 or less); one junior high school (grades 8-10), one senior high school (grades 10-12), and five high schools (grades 8-12). The socio-demographic characteristics of the participating schools did not differ significantly from those of the non-participating schools, with the exception of the neighbourhoods they served which had a higher percentage of visible minorities (42% versus 27%). All principals (n = 17) participated in the semi-structured qualitative interviews as well as 33 teacher/school informants (N = 50 informant interviews). Key informants were purposefully recruited by a delegated staff contact. Invitation letters were distributed in mailboxes and interested key informants returned consent forms to the school contact who then forwarded the information to the research team. Key informants were predominantly classroom teachers (n = 21) but also included PE specialists (n = 9), and cafeteria staff and home economics teachers (n = 3).

### Data collection

Semi-structured interviews were conducted individually with principals (n = 17) and key teachers/school informants (n = 33). Interviews were conducted in the 2010-2011 school year by two-trained research assistants. The interviews were 45 to 60 minutes long and consisted of broad open-ended questions with probes into emergent topics as they arose. Interviews started by asking the informants a number of background/demographic questions including questions about their position at the school, if applicable - courses and grades taught, and years of experience overall and at the current school. Questions related to the DPA and FBSS guidelines asked the informant to describe/discuss: a) their understanding and expectations of the guidelines, b) their thoughts about the guidelines, c) whether their school was implementing the guidelines as expected, d) how they were or were not implementing the guidelines; highlighting any examples (e.g. whether their school made any changes to implement the guidelines and if so what changes), e) the impact of the guidelines had on the school community (school, teachers, students, and parents), f) factors that facilitated or impeded implementation, and g) feedback or support received from the school community about the guidelines. Interviews were recorded digitally and researchers captured noteworthy aspects of the context or interview in supplementary field notes. Half of the interview targeted the DPA guidelines and the other half the FBSS guidelines. All informants provided written consent to be interviewed and received a gift card for participation ($15 Cdn). The designated school contact received a monetary incentive ($50 Cdn). Substitute teachers were provided to facilitate participation in the interviews.

### Data analysis

Digital recordings were transcribed verbatim and reviewed by the interviewers to ensure accuracy of the data. A constructivist grounded theory approach was used for coding [29,30]. It employs a less rigid application of grounded theory and explicitly acknowledges the researchers’ expertise and biases during the analysis process [30]. Two independent coders initially created a set of broad codes based upon the interview guide and research objectives. Transcripts were then coded line by line using an inductive method of open coding; whereby researchers allowed patterns and themes to emerge from the data [31]. To ensure the trustworthiness of the coding and interpretations of the data, the first five transcripts and every fifth transcript thereafter were coded in duplicate, and any discrepancies were discussed with a third researcher to reach consensus. Coding of transcripts continued until saturation. All data were coded in the NVivo 9 software (QRS International, 2010).

Concept maps were developed to reduce the qualitative data into meaningful concepts. This process was initially conducted separately for understanding the barriers and facilitators to the implementation of the guidelines. In the later stages, this process integrated knowledge of existing theoretical perspectives and it became apparent that the Diffusion of Innovations Model provided an organizing framework for presenting the emerging themes [32]. While this theoretical framework did not guide the original coding and analyses, it was used to summarize the data. Initial results were presented to key stakeholders (staff at the Ministries of Education (n = 2) and Health (n = 3) that had firsthand knowledge of these issues based on their interactions with school stakeholders related to guideline implementation) to check the validity of the interpretations and to gain further insights about our findings. This process also assessed convergence of the findings with other data sources collected by the provincial government and confirmed theoretical saturation as no new issues emerged in these discussions. This process served to ensure proper interpretation of our data.

## Results

### Perceived implementation

Implementation of the DPA and FBSS guidelines was reported by all schools but percentage of full implementation varied by guidelines (Table 1). Overall, the percentage of schools that perceived a full implementation of the DPA guidelines was higher in elementary schools than in middle/high schools; however elementary teacher/school informants reported much lower implementation than principals. In contrast, the percentage of schools that perceived full implementation of the FBSS guidelines was lower among elementary teacher/school informants than among middle/high school teacher/school informants. In middle/high schools, fewer principals thought they were meeting the FBSS guidelines than teacher/school informants (28.6% versus 42.8%); however, many indicated being close to complying with the guidelines.

**Table 1:** Percentage of informants who perceived their schools as fully implementing the Daily Physical Activity (DPA) and the Food and Beverage Sales in Schools (FBSS) guidelines

		**DPA guidelines**	**FBSS guidelines**
Principals	Elementary schools (n = 10)	90.0%	50.0%
	Middle & High schools (n = 7)	14.3%	28.6%
	All schools (N = 17)	58.8%	41.1%
Teacher/school informants	Elementary schools (n = 19)	42.9%	36.8%
	Middle & High schools (n = 14)	28.6%	42.8%
	All schools (N = 33)	36.4%	39.4%

(N = 50).

### Implementation styles/change

Schools implemented the DPA guidelines by taking either: [1] a prescriptive approach (requiring all students to participate) as they scheduled more PE/PA during instructional hours, added more PE classes, changed PE from a semester system to a full year class, scheduled jogging breaks, scheduled activity class before or after school, and/or incorporated classroom activity breaks; or [2] a non-prescriptive approach (providing more opportunities but not requiring) as they provided more PE/PA elective classes, expanded the intramural program, provided lunch hour games, allowed access to facilities outside of instructional time, added PA clubs (walking, running, and kilometre clubs), provided guidance on how to increase PA, encouraged students to be active through school announcements, sent newsletters, and/or had school assemblies with students. Higher grades were more likely to report implementing DPA in a non-prescriptive way.

To implement the FBSS guidelines, schools: changed content of vending machines, school store or canteen; changed vending machine supplier; made food healthier (e.g., chicken hot dogs with whole wheat buns); eliminated certain foods (hot dogs, French fries); eliminated vending machines, school stores, or canteens; required recipes be provided with bake sale items; changed portion sizes; eliminated donuts from staff meetings; cooked healthier recipes in home economics; changed the educational curriculum; eliminated outside food in the school; changed fundraising items; and eliminated classroom treats. Other less cited changes included: eliminating carbonated beverages (purchased or brought in from home), consulting with a nutritionist, sending newsletters to parents about HE and healthy recipes, having a healthy living week, asking concession stands to close when students are on field trips, prohibiting students from bringing money on school trips to prevent purchase of food and beverages, and starting an organic greenhouse and using the vegetables and fruits in the cafeteria.

### Barriers and facilitators to implementation

The emergent themes associated with the implementation of the DPA and FBSS guidelines are presented in Table 2 with illustrative quotes in Tables 3 and 4. With the exception of triability (e.g., the extent the which the innovation can be tried before deciding whether it is adopted/implemented), the themes were categorized by the characteristics of the Diffusion of Innovations Model [32]. The key innovation attributes described in the model (relative advantage compatibility, complexity, and observability) provide a relevant and robust framework to organize emergent themes for both the DPA and FBSS guidelines. The guidelines were mandated and thus triability did not emerge as a predominant theme. Contextual facilitators did emerge as a key theme which was added.

**Table 2:** Summary of the emerging themes for the Daily Physical Activity (DPA) and the Food and Beverage Sales in Schools (FBSS) guidelines

**DPA themes**	**FBSS themes**
**Relative advantage**	
• It’s better than what we were doing	• We needed this
• We had a better way of doing this before	• It would be better if we didn’t lose revenues
**Compatibility**	
• It fits our philosophy	• It fits my philosophy
• We like it versus it does not fit in my schedule	• The school community was on board
• There are favorable social norms	• There are favorable social norms
• Whose responsibility is it? We believe it is the family versus schools need to help those that don’t have	• Whose responsibility is it? We believe it is the family versus schools have a social responsibility to not profit from selling children unhealthy food
• This takes special skills	
• We (teachers) resent the top down approach taken for implementation	• We can’t follow it all the time, we need “one-time” exceptions
**Complexity**	
• We struggled with the lack of guidance	• We found it difficult to understand the scope of the guidelines
• We’re (elementary teachers) not clear what counts toward DPA	• We have to figure out what to do about decreasing profit margins
• We’re (elementary teachers) not clear that activities should be structured to count toward DPA	• We (teachers and Parent Advisory Council (PAC)) struggle to find suitable fundraising alternatives
• Evaluating implementation of DPA is hard	• We can be perceived as overstepping our boundaries as educators
• In higher grades it does not work as easily	• This pits the administrators against the parents
• We can’t meet all curriculum expectations versus it helps us meet our expectations	• We struggle with food insecurity
• We have to navigate cultural relevance	
• Our regional climate limits us	
**Facilitator**	
• Having ready-made provincial resources helped us (elementary teachers) with implementation	• Having provincial resources helped us with implementation
• This requires schools to have the appropriate resources	• Having access to a local nutritionist is helpful
• It is easier when physical education (PE) was a priority	• Having local suppliers that comply with the guidelines is necessary
• Having a PE specialist in elementary grades helps a lot	• Having mandated guidelines is useful ammunition for administrators
**Observability**	
• Some of us have noticed positive impacts (increased mental alertness and focus, improved academic performance, improved classroom behaviors, students enjoy being active, positive attitude shift toward physical activity, and increased positive student/teacher interactions)	• Some of us have noticed positive impacts (students/teachers are healthier, increased awareness about healthy eating, positive attitude shift toward healthy eating, and involved in more “green” initiatives)
	• We lost revenues
• It decreased teachers’ autonomy	• We had to reduce curricular and/or extracurricular activities
• It’s just more work for us (teachers & schools)	• We have noticed students selling unhealthy food
• We are encouraging students to falsify their physical activity data on their report cards	• More students leave school grounds at lunch – as a result they skip more classes and we are more concerned about their safety

**Table 3:** Sample quotes or explanations for the themes that emerged for the Daily Physical Activity (DPA) guidelines^*^

**Themes**	**Sample quotes**
**Relative advantage**	**It’s better than what we were doing** “..daily physical activity it seemed very overwhelming. But it didn’t take very long before it was “you know what, this is, this is great, this is something that should have been done a lot sooner.” (Elementary teacher)
	**We had a better way of doing this before “…**the Ministry needs to look at ways to get more PE specialists out in the schools… If we had a PE specialist and we had it .. all the students in the school would get a much higher level of quality out of their phys ed.” (Elementary teacher)
**Compatibility**	**It fits our philosophy “**I like the philosophy, …,a healthy society is absolutely part of what we should be encouraging and in education and setting the stage for a person’s healthy lifestyle and a vision of being healthy and active all their life…it’s all good .” (Elementary principal)
	**We like it “**I think it’s an awesome idea.” (middle/high school teacher) **versus it does not fit in my schedule** “Teachers would love to do it. But again, …their day will not allow it.” (Elementary principal)
	**There are favorable social norms** – Overwhelmingly, all informants talked positively about physical activity.
	**Whose responsibility is it? We believe it is the family “**I get that physical education should be part of our curriculum but I also don’t think it’s the school’s job solely to teach healthy living, I think it should also come from home … but telling the schools that they need to do it, yeah, … I don’t necessarily agree with it.” (Elementary teacher) **versus schools need to help those that don’t have** “…these kids really need that time … because I know a lot of them do not get outside of school exercise. Like they’re not in soccer, they’re not into dance; they’re not into hockey or whatever.” (Elementary teacher)
	**This takes special skills “…**even though, everyone to my knowledge in Canada has to take a PE methodology course, there are very very many people…teaching in elementary school who are uncomfortable with PE, they’re uncomfortable with getting the students to do activities, and gym results in dodge ball for a lot of the year.” (Elementary teacher)
	**We (teachers) resent the top down approach taken for implementation** “…they think the idea (DPA) is good but they’re questioning the way it’s being implemented, yeah, more so than with the food.” (High school teacher)
**Complexity**	**We struggled with the lack of guidance** “I think the schools were looking for some guidance from the districts, the districts dumped it back to the school…it would be nice to have a specific guideline saying ‘this is what you’re doing, this is how you do it… different schools [in this school district] are having to come up with … different models to implement in theory the program that could be just implemented across the board. So I would say, why isn’t the district just coming up with a common model that everybody is supposed to follow and you just do it?” (Middle/high school principal)
	**We’re (elementary teachers) not clear what counts toward DPA –** How much physical activity students need to accumulate? Is it 15, 20, 30, and 60 minutes per day or 10 minutes in addition to PE. Can DPA be accumulated at recess or lunch time and what about non-instructional hours? Does only vigorous physical activity counts toward DPA but what about strength training activities?
	**We’re (elementary teachers) not clear that activities should be structured to count toward DPA** “I know that some schools feel that the recess lunch time should be part and parcel of the DPA just for management reasons. But I mean you can’t guarantee that your kids are going to be active out there at recess and lunch. Some of them might just stand there and be, you know, bystanders.” (Elementary teacher)
	**Evaluating implementation of DPA is hard “**We’re meeting the requirements of having it on the report card, and encouraging parents to work with the kids to meet it…but if you asked me to pull out the actual guidelines and show you step by step how we’re implementing – no.” (Middle/high school principal)
	**In higher grades it does not work as easily “…**it fits into an elementary timetable… in an elementary school you can add those minutes in; much harder to do in a secondary where they are going from teacher to teacher. And every teacher is mandated to spend so many minutes with their kids and now you’ve got 30 minutes added in. Elementary: okay, secondary: difficult. “ (Middle/high school teacher)
	**We can’t meet all curriculum expectations** “…meeting the language arts curriculum, meeting the math curriculum requirements, social studies, science, health education, PE, computer technology, it’s all of it and each year the Ministry gives us one more piece. Well there aren’t enough minutes in a day; we’ve run out, we ran out years ago.” (Elementary principal) **versus it helps us meet our expectations** “I don’t see that as taking away from other parts of their curriculum to do this, I see this as all one curriculum…I would posit that by doing your 30 minutes of exercise you’ve actually helped your social studies program: kids are more primed to learn, they’re more apt to spend time on it” (Elementary principal)
	**We have to navigate cultural relevance** “…our greatest challenges… is with our families where English is not the predominant language in the home. And working with those families to help them understand that it’s really important for their kids to get out and be active outside of school is a challenge because they might not hold that same value culturally…Their values might be more on academics.” (Elementary principal)
	**Our regional climate limits us “**I mean up here in the north it gets cold early in the season so they can’t go outside everyday right, it’s just not necessarily practical…I mean in the lower mainland I guess they can go outside and do some exercises, activities, go for a walk but whatever, whatever, you know, the teacher can or wants to do with them but we have, we don’t always have outside as an option up here.” (Middle/high school teacher)
**Facilitator**	**Having readymade provincial resources helped us (elementary teacher) with implementation** “I think by way of receiving materials (from Action Schools BC) like that, and then the workshop that we got from the Action Schools – I think that, all that kind of stuff helps, yeah.” (Elementary teacher)
	**This requires schools to have the appropriate resources** (gymnasium, nearby park or community center, large outdoor field or playground area) “This school being quite small they were able to have access to the gym a lot more than other schools – like I was at another school… they had to share all their PE classes with like two or three or four other classes. So they have the benefit here of not only having the gym to themselves…they’re able to sign up for other extra gym periods which definitely helps.” (Elementary principal)
	**It is easier when physical education (PE) was a priority –** Meaning if the schools had more scheduled PE in place, many PE electives, or many PA classes it facilitated implementation.
	**Having a PE specialist in elementary grades helps a lot “(**What helps implementation?)…I think having somebody that’s a real champion when you look at (PE Specialist name), you know, when she has the kids I mean she works them pretty hard during PE time there’s no sloughing off there. You know the kids are, they’re put to task and they seem to enjoy what they’re doing and that’s the nice thing about having one person pretty well that does all of the PE at the elementary level.” (Elementary principal 0936)
**Observability**	**Some of us have noticed positive impacts** (mental alertness and focus, improved academic performance, improved classroom behaviors, student enjoy being active, attitudes shift toward physical activity, and increased positive student/teacher interactions) “I thought, I would be fighting up-against a wall to get this done; and the students love it..they crave it. I like ‘okay, yup, yup, what are we doing for fitness today?’ they want to be in shape and they know it’s important…and there’s no complaint, there’s nothing” (Elementary teacher) “So it has me thinking during the school day. How can I get my kids more active? … it’s good to have that in the back of my mind knowing that … each day, I have to think of how can I get my kids moving.” (Elementary teacher)
	**It decreased teacher’s autonomy** – Elementary teachers have less freedom to structure their daily activities.
	**It’s just more work for us (teachers & schools) “**The videos are fun. But, uh, no, it’s just, it’s an added stress. Yeah, I’m just cranky about the whole dumb thing [laughs].” (Elementary teacher)
	**We are encouraging high school students to falsify their physical activity data on their report cards** “The kids have to write in their booklets once a week for 10 minutes and they write down everything they did all week. So, they make it up it’s not worth any marks. It’s a joke. So, but it’s a good start, they’re aware. They’re forced to do it. They can reflect on it and some of them take it seriously but others don’t… If DPA had some teeth then those are the ones that would benefit. And it’s not just students who are overweight…it’s the students who are…skinny, but you know they’re just not healthy.” (Middle/high school teacher)

* All quotes are in parentheses and clarifying text is not.

**Table 4:** Sample quotes or explanations for the themes that emerged for the Food and Beverage Sales in Schools (FBSS) guidelines

**Themes**	**Sample quotes**
**Relative advantage**	**We needed this “**Well I think it’s, it’s late in coming but it’s about time, yeah” (Middle/high school teacher)
	**It would be better if we didn’t lose revenue “**“I understand the meaning of it and…the students do need to eat healthier, but…it didn’t change, like the desired effect was for students to eat healthier but the majority of them it didn’t change much, it just sent the money elsewhere, so. I don’t know what the answer is but, it was a huge negative impact and it really, it really affected us quite a bit.” (Middle/high principal)
**Compatibility**	**It fits with my philosophy “**I personally I value what the province is doing, I understand it and I think they’re right minded with trying to change behavior patterns with the young through the schools so I’m supportive to that in the philosophy that it has.” (Elementary principal)
	**The school community was on board** “Here they’ve responded well. I think everybody seems to be on board.” (Elementary principal)
	**There are favorable social norms** “Mostly students, all the teachers are on board with it, cause we all, you know, I think healthy eating is definitely one thing that – well obesity is a problem right with, all across north America… we’re trying to promote healthy living, so.” (High school teacher)
	**Whose responsibility is it? We believe it is the family “**Well personally I think that it’s the job of parents to be properly feeding and clothing their kids.” (Principal 1536) **versus schools have a social responsibility to not profit from selling children unhealthy food** “Personally I don’t believe that we should be selling pop in schools, I don’t believe we should be selling chocolate bars in schools. I think that those are items that if parents choose to have their children eating them that’s their own choice, but I don’t think that we should be promoting or profiting from the sales of anything that isn’t healthy.” (Elementary principal)
	**We can’t follow it all the time; we need “one time exceptions” “**So what was at the grad barbecue? Well, there was regular coke and stuff like that. They wouldn’t fall under the guidelines, so. We just kind of ignore the guidelines and because, if we brought in juices and stuff like that, probably wouldn’t be drank at an event like that.” (Middle/high school principal) and“There are times when we do fundraising, obviously a pizza sale one day for a trip…it’s not following the Guidelines, but it’s for fundraising for the school.” (Elementary principal)
**Complexity**	**We found it difficult to understand the scope of the guidelines** (just vending or all food served) **and where they apply** (fundraising activities, activities organized by Parent Advisory Council, bake sale items, classroom treats, outside school hours activities, advertizing in school, accepting sponsorship from companies, and accepting incentives from vending machines company)
	**We have to figure out what to do about decreasing profit margins “**Well it’d be revenue from the vending machines, but also the cafeteria. Cause they can’t sell – cause like a lot of the junk food were good money makers… Higher margin of profit, whereas quality food, the profit margin is not there.” (Middle/high school principal)
	**We (teachers and Parent Advisory Council (PAC) struggle to find suitable fundraising alternatives “** So as I said, for our PAC (Parent Advisory Council)…certainly they’ve communicated they find it somewhat…restricted in what they can sell (for fundraising).” (Elementary principal)
	**We can be perceived as overstepping our boundaries as educators** “Looking at kids’ lunches and recognizing the things that are healthy and commenting on it, … that gets the ire of some parents up because you’re embarrassing my children when you’re talking about their lunch. You shouldn’t be doing that. If you want to talk to me as the parent about it, that’s one thing, but don’t talk to the kid.” (Elementary teacher)
	**This pits the administrators against the parents** “And we were greeted by a big cheer…the parents thought “yeah, great”. And so… we still have parents who come every day and drop off McDonald’s lunches and stuff, you know, we were saying, we can’t stop them from doing that, but we’d say “you can’t bring pop, you have to bring something else for them to drink.” And most parents would go “Oh, OK, I didn’t know. Yeah, fine.” “And it was going OK for awhile, until one of our PAC executive members…took exception to the pop-free rule, saying that it…infringed upon parental rights, because it was the parent’s right to decide what the children ate, what they put in their child’s lunches… we were now putting a child in a compromised position… the parent might be in a…situation where they have nothing else at home to feed their child….so we had to [sigh] for the sake of, I don’t know, relationships…repeal that policy, and we sat down together – administration and PAC to come up with a statement that we all agreed upon and then sent it out to the community” (Elementary principal)
	**We struggled with food insecurity** “We’re an inner city school…in some cases feeding kids is more important.” (Elementary principal)
**Facilitator**	**Having provincial resources helped us with implementation** (e.g., booklet provided by the Ministry of Education, website, and the Fruit and Vegetable program)
	**Having access to a local nutritionist is helpful “**I mean, we’re lucky in the sense that food and beverage is with the cafeteria program. The district does have a director that’s in charge of all programs, so she (district nutritionist) oversees, even school stores, what’s being purchased … I mean that’s beneficial so we have to follow those guidelines, just like the chef has to order specific things, sometimes not happy about it, but to follow those guidelines, she does.” (Middle/high school principal)
	**Having local suppliers that comply with the guidelines is necessary “**Finding a vendor, like this vendor is now out of XXX,… we get a new delivery person every month …they know what they’re supposed to put in [the vending machine], but they’re visiting, I don’t know how many sites a day, and “Oh, I don’t have any of this stuff left, so I just want to fill the hole, so I put in Coke Zero” or whatever…We probably had to phone 10 times during the year…“Get that guy back here and tell him to pull out that Coke.” You know, that’s the last thing I wanna have to waste time on… So, we’ve had some problems in that general area.“ (High school principal)
	**Having mandated guidelines is a useful ammunition for administrators** “Having the (FBSS) guidelines is helpful because it gives you sort of that ammunition behind you to say ‘well there’s a provincial guideline and it’s expected.” (Middle/high school principal)
**Observability**	**Some of us have noticed positive impacts** (students/teachers are healthier, increased awareness about healthy eating, positive attitude shift toward health eating, and involved in more “green” initiatives) **“**Oh definitely so, yeah. I found this (the FBSS guidelines) made a difference to kid’s attitudes and especially their behaviour, you know, especially with a lot less sugar.” (Elementary principal)
	**We lost revenues** – without being prompted many middle/high school principals noted a revenue loss of $10,000 to $45,000.
	**We had to reduce curricular and/or extracurricular activities “…**it’s great to be promoting healthy food, on the one hand it’s, you know, our athletic program half the funding we used to have for running our athletic program was coming from the profits.” (High school principal)
	**We have noticed students selling unhealthy food** – entrepreneurial student took upon themselves to “illegally” sell less healthy food items.
	**More students leave school grounds at lunch – as result they skip more classes and we are more concerned about their safety** “If I had the vending machines capability, I bet I would keep another 50, 60, 100 kids on site, which I would prefer because it’s when they go off site that I lose them, that’s where they skip, or get into trouble, or decide to buy something they shouldn’t be buying. And if I can keep them on-site I have more, for lack of a better word, control of what they’re doing with their lunch breaks.” (Middle/high school principal)

* All quotes are in parentheses and clarifying text is not.

#### Relative advantage

In general, informants had a positive opinion of both guidelines relative to the status quo (“it’s better than what we were doing” for the DPA guidelines and “we needed this” for FBSS). However, some elementary school informants, who were in the school system prior to budget cuts that eliminated PE specialists in elementary schools in the late 1980’s and early 90’s thought they “had a better way of doing this before” when they had PE specialists. The irony of this was discussed in the context of the DPA guidelines. In contrast, while the FBSS guidelines were perceived as positive from a ‘health’ perspective, their advantage were often tempered by their potential negative impact on school revenues.

#### Compatibility

Both guidelines were perceived to be compatible with schools’ or teachers’ expectations of what the school learning environment should provide (“it fits our philosophy” and “we like it” for DPA guidelines and “it fits my philosophy” and “the school community was on board” for FBSS guidelines). Most statements about the DPA guidelines focused on how this fits with “us”, the “school” or “teachers”, while the responses about the FBSS guidelines focussed more on how they affect “us” as “individuals”. Informants talked positively about the importance of PA and HE suggesting strong favorable social norms about these behaviours which in some cases translated into positive feelings toward the guidelines but not always. For both guidelines, some informants questioned whether schools or parents should be responsible or solely responsible for providing PA and HE opportunities. Others felt schools had a social responsibility to address these issues (“schools need to help those that don’t have” for the DPA guidelines and “schools have a social responsibility to not profit from selling children unhealthy food” for the FBSS guidelines). Finally, compatibility issues specific to DPA included the difficulty in fitting DPA into the schedule, lacking skills to provide more PA and resenting the top down approach taken in mandating the DPA guidelines. For the FBSS guidelines, some informants felt strongly about the need to allow for “one-time exceptions” to ensure it fits with how schools function.

#### Complexity

Many of the complexity issues revolved around understanding of the guidelines. For the DPA guidelines, many struggled with the lack of direction provided in the guidelines; what counted toward DPA and how activities should be structured to count toward DPA. For the FBSS guidelines, many were not sure about the scope of the guidelines; whether they applied only to vending machines or to all food and beverages and also when (within school hours) and where (outside school events) the guidelines applied.

Other complexity issues revolved around the barriers teachers or schools encountered when they tried to fully implement the guidelines. For the DPA guidelines, informants from higher grades (where reporting on DPA by students was required) struggled to report how students met their PA requirements on report cards. In contrast, informants from lower grades struggled with meeting all curriculum expectations. Schools with a large immigrant population talked about the need to address the value of PA to ensure implementation of the DPA guidelines in this population. Schools where the weather tended to be more extreme talked about their struggle to implement the DPA guidelines in inclement weather.

For the FBSS guidelines, schools struggled with maintaining profit margins and finding suitable fundraising alternatives. In addition, some schools had encountered problems when their teachers eagerly embraced the guidelines but parents perceived that they had overstepped educational boundaries by talking to the children about or forbidding some food. Finally, in disadvantaged schools, where food security was a widespread concern, administrators struggled to implement the guidelines while also trying to feed their disadvantaged students.

#### Facilitators

Appropriate resources and support were considered key facilitators to the implementation of both guidelines. For the DPA guidelines, this included having ready-made provincially available resources (Action Schools! BC), school resources (gymnasium, nearby park or community center, large outdoor field or playground area), and a PE specialist in their school (elementary grades). Implementation was also easier in schools where PE was a priority prior to the implementation of the guidelines. In the context of the FBSS guidelines, having provincial resources that were developed to support implementation (e.g. Brand Name Foodlist) as well as existing programs available were seen as facilitative. In addition, having a nutritionist available for consultation (a resource added with the launch of the FBSS guidelines through Dietitians Services) and having local suppliers that complied with the guidelines facilitated implementation. Interestingly, the top-down approach to enact the school guidelines, which was seen as problematic by some, was perceived by one administrator as helpful; it helped to create positive changes in the school without the administrator taking personal blame.

#### Observability

Positive impacts were observed as a result of implementing the guidelines (see Table 2). Unintended consequences were also noted for the DPA guidelines, including teachers feeling they had less autonomy over their schedules and increased workload through direct delivery at the elementary school level and through the responsibility of tracking and documenting student DPA at the high school level. Unintended observable consequences of the FBSS guidelines implementation included: loss of revenue from food/beverage sales, fundraising activities and bake sales; reduced funds for curricular and/or extracurricular activities (field trips, music program, social assistance program, athletic program, sporting events, and others); selling of unhealthy food and beverages by “entrepreneurial students”; and more students leaving school grounds at lunch time which then resulted in students being late to class after their lunch break, skipping classes, or increased concerns about student safety.

## Discussion

Our study provided an in-depth analysis of the factors that influenced implementation of school-based PA and nutrition guidelines in BC, Canada. Implementation of the DPA and FBSS guidelines was influenced by perceptions that the guidelines: were relatively advantageous compared to status quo, were compatible with school mandates and teaching philosophies, were complex to understand and implement, and had observable positive impacts. A number of contextual factors including availability of resources facilitated implementation; however, tremendous variability was observed across schools in terms of the factors identified as influencing implementation. Interestingly, the target of the guidelines also contributed to the variability reported in the effectiveness of implementation between guidelines and schools. The FBSS guidelines targeted the school environment and thus its predominant impact was at the school level, including the Parent Advisory Council (PAC) in some cases. In contrast, the DPA guidelines targeted both teachers and schools; as teachers were responsible for the delivery of PA in the context of the classroom in lower grades and schools were responsible for documenting how students met the DPA requirements in higher grades.

Our findings support the findings of similar studies of the U.S. school wellness policy. These studies found similar barriers to implementation such as: revenue loss, competing demands, lack of resources, support from the school community, difficulty in finding fundraising alternatives, and decreased resources for curricular or extracurricular activities [5,19,22,24]. Some of our findings are perhaps unique to the Canadian context and other countries that do not have subsidized federal school meal/breakfast programs [33]. For example, addressing food insecurity is more complex when schools are not provided with infrastructure support or funds to subsidize school meals. In addition, schools cannot count on the subsidized school meal to maintain cafeteria revenues when modifying their “A La Carte” offerings. This has been found to offset anticipated revenue loss in some U.S. schools, where school meals are subsidized [34]. Our findings may also be unique because other countries have focused on PE- rather than PA-related school policies. For example, understanding how the DPA guidelines are implemented outside of PE and dealing with increased elementary teacher workloads are highly relevant themes in the context of DPA but not PE. Finally, some findings of the current study have not been documented in previous studies but may apply to similar policies and contexts, including statements about: needing one-time exceptions for HE policies, shifting the responsibility from schools to parents, navigating cultural relevance, having more students leave school grounds (high schools only), and having more green initiatives integrated in fundraising activities.

Our study is one of the first qualitative studies to use the key attributes of the Diffusion of Innovations Model to organize the factors associated with implementation of school-based PA and nutrition policies/guidelines [32]. With the exception of triability, the key innovation attributes described in the model (relative advantage, compatibility, complexity, and observability) provide a relevant and robust framework to organize emergent themes for both the DPA and FBSS guidelines. Most importantly, this framework provides a useful structure for planning and developing strategies to improve the implementation of school-based policies/guidelines.

Strengthening the *relative advantage* of DPA guidelines may require: increasing access to PE specialists at the school or district level, providing training, and sharing the evidence linking PA to improved cognitive functioning and academic performance in schools [14]. In addition, sharing the evidence may also enhance their perceived *compatibility* as some informants felt parents rather than schools should take responsibility for PA. Addressing *complexity* may require: providing more direction to schools on how to meet the guidelines, sharing models of successful implementation, supporting ethnically diverse schools that lack parental support for the guidelines, and developing an infrastructure to facilitate tracking and documentation in higher grades. *Observability* is an interesting characteristic to address as teachers can observe the positive impact of DPA on students’ health or academic performance; however, enhancing the benefits to teachers also seems important given concerns of increased workload and school expectations.

Communicating the health related evdence [6-8,11-13,17,18] in support of the FBSS guidelines appears important for bolstering their *relative advantage* and offsetting mixed feelings associated with their potential impact on school revenues. Similar to the DPA guidelines, perceived *compatibility* may also be improved by communicating the evidence linking HE with academic performance [15,16]. Issues raised under c*omplexity* highlighted the need to: educate teachers on how to talk sensitively about HE without usurping parental authority, find suitable fundraising alternatives to minimize conflicts with PAC, minimize revenue loss, and ensure disadvantaged students are not negatively impacted by the guidelines. *Observability* can be strengthened by implementing strategies to: prevent or subsidize loss of revenues, reduce the number of students leaving school grounds at lunch time, and prevent the entrepreneurial students from selling unhealthy food.

Our findings also uncovered the contextual factors that facilitated implementation of mandated guidelines and highlighted the importance of available resources as a common facilitator. The implementation of the DPA guidelines in BC was preceded by the large scale dissemination of Action Schools! BC, a comprehensive whole school approach to increasing PA opportunities across six school action zones (including PE and the classroom) [35]. The dissemination of the initiative began four years before the DPA guidelines were implemented and provided schools and teachers with planning tools, equipment, training, on-going technical support and a suite of better practice resources that were developed for the school setting and ready to use [35]. Participants specifically highlighted this as a facilitator. In the absence of such infrastructure, it is likely that other jurisdictions attempting to implement similar guidelines may be faced with more barriers than we observed. Action Schools! BC integrated HE in 2008 which once again provided the same supports to schools and teachers to modify their school food environment.

Finally, considerable variability was observed in terms of the strategies employed to implement these guidelines. The extent to which some strategies (e.g., using a prescriptive or non-prescriptive approach to implement the DPA guideline) are more effective than other ones remain unknown and deserves further investigation. In addition, we do not know whether the DPA and FBSS guidelines significantly improved the school environment. Many informants indicated the school PA and food environment improved with the implementation of these guidelines; however, much more work needs to be done to uncover whether policy strategies alone or combined with other approaches will help reverse the current childhood obesity epidemic.

## Conclusions

In conclusion, emerging evidence suggests school-based policies targeting the school PA and food environment can influence student behaviors [4,9-13]. The enactment of mandated guidelines/policies is considered an essential step in changing the school PA and food environment. However, effective implementation is critical to success. Policy makers need to: monitor whether schools are able to implement the guidelines, provide support to schools struggling with implementation, and document whether the guidelines are influencing students’ behaviors as intended. The Diffusion of Innovations model provides a useful framework for understanding the factors that impede or facilitate implementation of guidelines in schools.

## Abbreviations

DPA: Daily physical activity; FBSS: Food and beverage sales in schools; HE: Healthy eating; PA: Physical activity; PAC: Parent advisory council; PE: Physical education.

## Competing interests

We the authors, have no financial or non-financial competing interests with the content of this manuscript.

## Authors’ contributions

LM is the principal investigator on a Canadian Institutes of Health Research grant, wrote the grant, supervised and trained all research staff, developed the data collection and analysis protocols, analyzed the data, drafted the manuscript, and finalized the manuscript. DN was a research assistant on the grant, collected data, managed the coding of the qualitative data, drafted sections of the manuscript, and critically reviewed the manuscript, and approved the manuscript. PJN is a co-investigator on the grant, provided input in the grant and development of the data collection and analysis protocols, critically reviewed the manuscript, and approved the manuscript. All authors read and approved the final manuscript.
